# Impact of New Systemic Treatment and Radiotherapy in Melanoma Patients with Leptomeningeal Metastases

**DOI:** 10.3390/cancers12092635

**Published:** 2020-09-16

**Authors:** Pauline Tétu, Lila Sirven-Villaros, Stefania Cuzzubbo, Renata Ursu, Barouyr Baroudjian, Julie Delyon, François Nataf, Constance De Margerie-Mellon, Clara Allayous, Wendy Lefevre, Antoine F. Carpentier, Céleste Lebbé

**Affiliations:** 1 Assitance Publique des Hôpitaux de Paris Dermatology, Department of Dermatology, Hôpital Saint-Louis, 75010 Paris, France; barouyr.baroudjian@aphp.fr (B.B.); julie.delyon-ext@aphp.fr (J.D.); clara.allayous@aphp.fr (C.A.); wendy.lefevre-ext@aphp.fr (W.L.); celeste.lebbe@aphp.fr (C.L.); 2 INSERM U976, Paris 7 Diderot University, 75475 Paris, France; 3 Service de Neurologie, Assistance Publique-Hôpitaux de Paris (AP-HP), Hôpital Saint-Louis, 75010 Paris, France; lila.sirvenvillaros@aphp.fr (L.S.-V.); stefania.cuzzubbo@inserm.fr (S.C.); renata.ursu@aphp.fr (R.U.); antoine.carpentier@aphp.fr (A.F.C.); 4 Department of Neurosurgery, Groupement Hospitalo-Universitaire-PARIS, Hôpital Sainte-Anne Paris, 75014 Paris, France; f.nataf@ghu-paris.fr; 5 INSERM UMR 1149, Université de Paris, F-75018 Paris, France; constance.de-margerie@aphp.fr; 6 Radiology Department, Assistance Publique-Hôpitaux de Paris, Hôpital Saint-Louis, F-75010 Paris, France

**Keywords:** leptomeningeal melanoma, survival, radiotherapy, immunotherapy, targeted therapy

## Abstract

**Simple Summary:**

Although the recent years have seen incredible progress in the treatment of advanced melanoma including brain metastases, the survival of melanoma patients with leptomeningeal disease (LM) is still poor. There are currently only a handful of reports suggesting that BRAF and MEK inhibitors, or immune checkpoint inhibitors, may induce survival benefit in advanced melanoma patients with LM. The objective of this study was to gain a better understanding of patients, disease characteristics, and therapeutics interventions in LM melanoma patients in the era of new systemic treatment and radiosurgery. This study demonstrated that new treatment modalities appear to be promising new treatment options for melanoma LM, and need to be tested in larger prospective studies. A subset of patients are long-term survivors, and factors associated with improved survival such as low serum LDH level may be identified.

**Abstract:**

Importance: Few data are available on patients with leptomeningeal disease (LM) from melanoma treated with new systemic therapies. Objective: To gain a better understanding of patients, disease characteristics, and therapeutic interventions in melanoma patients with LM in the era of new systemic treatment. Design: Clinical characteristics, treatments, and survival of melanoma patients diagnosed with LM, isolated or associated with brain metastases, were collected. The Cox regression model assessed the influence of patient and melanoma characteristics on survival. Setting: Monocentric, retrospective, real-life cohort of patients with LM from melanoma. Participants: All patients followed up at Saint-Louis University Hospital and diagnosed with LM between December 2013 and February 2020 were included. For each patient identified, a central review by dermato-oncologist and neuro-oncologist experts was performed to confirm the diagnosis of LM. Exposure: Impact of new systemic therapies and radiotherapy. Results: Among the 452 advanced melanoma patients followed at St Louis Hospital between 2013 and 2020, 41 patients with LM from melanoma were identified. Among them, 29 patients with a diagnosis of LM “confirmed” or “probable” after central neuro-oncologists reviewing were included. Nineteen patients had known melanoma brain metastases at LM diagnosis. Among the 27 patients treated with systemic therapy, 17 patients were treated with immunotherapy, 5 patients received targeted therapy, 1 was treated with chemotherapy, and 4 patients were treated with anti-PD-1 in combination with BRAF inhibitor. The median overall survival (OS) from LM diagnosis was 5.1 months. Median OS was 7.1 months for the 9 patients receiving systemic therapy combined with radiotherapy, and 3.2 months for the 20 patients not receiving combined radiotherapy. Elevated serum lactate dehydrogenase (LDH) (HR 1.44, 95% CI 1.09–1.90, *p* < 0.01) and presence of neurological symptoms at LM diagnosis (HR 2.96, 95% CI 1.25–6.99, *p* = 0.01) were associated with poor survival. At the time of data analysis, five patients were still alive with a median follow-up of 47.4 months and had persistent complete response. Conclusion: Targeted therapy and immunotherapy are promising new treatment options in LM from melanoma that can increase overall survival, and may induce long lasting remission in some patients.

## 1. Introduction

Leptomeningeal disease (LM) is one of the most debilitating complications occurring in metastatic melanoma. LM typically affects about 5% of advanced melanoma patients [1] and a link has been suggested between the presence of melanoma brain metastases (MBM) and the development of LM, with up to 19% of patients having concomitant parenchymal and leptomeningeal metastases [2]. LM is traditionally associated with very poor outcomes, with a historical median survival of only 4–10 weeks [2,3]. Until recently, whole-brain radiation therapy (WBRT) and chemotherapy (systemic or intra-thecal) were the only available therapeutic options in LM melanoma patients with poor survival benefits [2,3,4,5]. The recent years have seen incredible progress in the treatment of advanced melanoma, including MBM, with the development of targeted therapy and immunotherapy [6,7,8,9,10]. However, metastatic melanoma patients with evidence of LM were excluded from all these clinical trials [6,7,8,9,10]. There are currently only a handful of reports suggesting that BRAF and MEK inhibitors, or immune checkpoint inhibitors, may induce survival benefit in advanced melanoma patients with LM [11,12,13]. Focal radiotherapy could be used for the treatment of limited macroscopic leptomeningeal disease, whereas the use of WBRT is decreasing owing to poor survival benefit and radiation-induced cognitive toxicity [14]. Combined radiotherapy with new systemic therapies has shown a significant survival benefit in MBM patients [15], but no study has investigated the efficacy of combined strategy in the field of LM.

The objective of this study was to gain a better understanding of patients, disease characteristics, and therapeutics interventions in LM melanoma patients in the era of new systemic treatment and radiosurgery.

## 2. Material and Methods

### 2.1. Patients

Patients included in this study were identified via MelBase, a French clinical database with a biobank dedicated to the prospective follow-up of adult patients with advanced melanoma. This observational cohort included standardized questionnaires completed prospectively by the investigator at inclusion and throughout disease course. The MelBase protocol was approved by French ethics committee (CPP Ile-de-france XI, n°12027, 2012) and registered in the NIH clinical trials database (NCT02828202, https://clinicaltrials.gov/ct2/show/NCT02828202). Written informed consent was obtained from all patients.

All patients within Melbase followed up at Saint-Louis University Hospital and diagnosed with LM between December 2013 and February 2020 were included. For each patient with LM from melanoma identified via Melbase, a central review by dermato-oncologist and neuro-oncologist experts was performed to confirm the diagnosis. The diagnosis of LM was established by MRI and clinical symptoms according to European Association of Neuro-Oncology (EANO) and European Society for Medical Oncology (ESMO) leptomeningeal metastases guidelines and cerebrospinal fluid analysis (CSF) when available [16]. Only patients with “confirmed” or “probable” LM after central neuro-oncologists reviewing of MRI were included.

### 2.2. Data Collection

Data were collected retrospectively, including: sex, age, Eastern Cooperative Oncology Group (ECOG), serum lactate dehydrogenase level (LDH), BRAF and NRAS mutation status, primary melanoma site, presence of MBM, number and volume (< or >2 cm diameter) of MBM, use of corticosteroids, neurological symptoms at LM diagnosis, number of previous systemic treatment lines, CSF results, date of LM diagnosis, date of metastatic melanoma diagnosis, date of primary melanoma diagnosis, therapies received before and after LM diagnosis (including systemic therapies and radiotherapy), and date of death or last follow-up.

Patients were classified in two groups independently of systemic treatment received: receiving combined radiotherapy (cRT group) or not (no-cRT group). RT was considered combined if delivered from 30 days before the first systemic therapy dose to treat LM until 30 days after the first dose of the same therapy line. All lesions treated with RT, within a timing of more than 30 days from the start of the same therapy line, were included in the no-cRT group. The cRT was performed according to local practices and included WBRT alone, stereotactic radiosurgery (SRS) alone, or both.

### 2.3. Endpoints

In both groups, date of LM diagnosis was considered as baseline. The primary endpoint was OS, defined as the time from baseline to death from any cause or end of follow-up. The secondary endpoints were progression-free survival (PFS) and objective response rate (ORR). PFS is defined as time from baseline to clinical or radiological progression or death from any cause. “Clinical or radiological progression” was defined by the presence of new lesion or progression of existing lesion on clinical and radiological evaluation during the prospective follow-up, according to Response Evaluation Criteria in Solid Tumours (RECIST 1.1.) (https://project.eortc.org/recist/wp-content/uploads/sites/4/2015/03/RECISTGuidelines.pdf) Radiological evaluation included brain MRI (magnetic resonance imaging) and total body imaging (PET-scan or CT scan), and was performed every three months. ORR was defined as the proportion of patients with complete response (CR) or partial response (PR) as best overall response. Intra-cranial (IC) and extra-cranial (EC) ORR were evaluated by central dermato-oncologist and neuro-oncologist review.

### 2.4. Statistical Analysis

The Kaplan-Meier method was used to estimate the OS and PFS from the date of LM diagnosis. Cox proportional hazards regression was used to assess the association between covariates of interest and OS. Statistical analysis was performed using R studio version 3.5.2. (https://cran.r-project.org/bin/windows/base/old/3.5.2/).

## 3. Results

### 3.1. Patients and Disease Characteristics at LM Diagnosis

Among the 452 patients enrolled in our center between 2013 and 2020, 41 patients with LM from melanoma and 148 patients with MBM were identified via Melbase. After central neuro-oncologist and dermato-oncologist review, 12 patients with “possible” diagnosis of LM were excluded. Twenty-nine patients with “confirmed” and “probable” LM were included in the analysis.

Patient and disease characteristics at time of diagnosis of LM are summarized in Table 1.

The median age was 55 years (range 50–67) and 18 patients (62%) were male. At diagnosis of LM, 75% of patients had ECOG 0 or 1 and 69% of patients had normal LDH level. Approximatively 45% of patients (*n* = 13) had BRAF mutated melanoma. Fourteen (48%) patients were naïve of systemic treatment at LM diagnosis.

Median time from diagnosis of primary melanoma to LM was 31.5 months (range 15.5–39.4) and from diagnosis of advanced melanoma (unresectable stage III or stage IV) to LM was 5.2 months (range 1.2–6.8).

For ten patients (34%), LM was the first presentation of central nervous system (CNS) disease, while most of the patients (66%) had a history of known MBM prior to LM diagnosis. Median time from diagnosis of MBM to LM was 2.4 months (range 0–5.6). Seventeen patients (58%) had received previous CNS radiation and five patients (17%) had had previous surgery for MBM.

### 3.2. Diagnosis of LM

Fifteen patients (52%) presented neurological symptoms at LM diagnosis. The most common LM symptoms were headache (34%) and nausea/vomiting (14%). CSF cytology was performed in only two patients and tumor cells were identified in only one case. The diagnosis of LM was based on MRI alone in 28 patients (97%) and on the combination of positive CSF cytology and MRI in one patient. According to the EANO-ESMO guidelines, the diagnosis of LM after certified neuro-oncologist central review was “confirmed” in 1 patient and “probable” in 28 patients. Nineteen patients (66%) had known melanoma brain metastases at LM diagnosis.

### 3.3. Systemic Treatments and Radiation Therapy for LM

**Figure 1:**
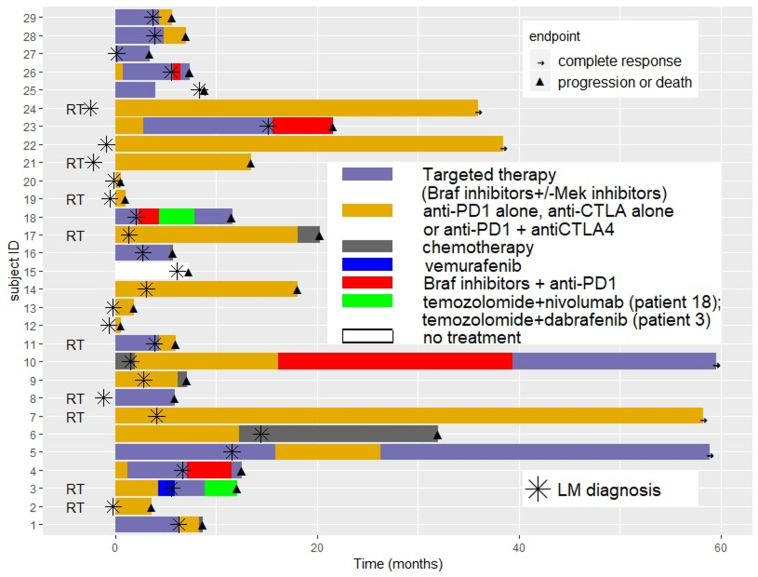
Sequence of systemic treatment and radiotherapy received before and after leptomeningeal disease diagnosis for each patient.

The sequence of systemic treatment and RT received before and after LM diagnosis for each patient is presented in Figure 1.

At time of LM diagnosis, 48% of patients (*N* = 14) were naïve of systemic treatment, 21% (*N* = 6) had received one previous systemic line, and 31% (*N* = 9) two or more previous systemic lines. Among patients receiving systemic therapies at LM diagnosis, treatment was targeted therapy for 10 patients, immunotherapy for 4 patients, and chemotherapy for 1 patient. Four patients continued to receive the same systemic treatment line after the diagnosis of LM than as before.

Twenty-seven patients (93%) received treatment (systemic treatment or radiation therapy) after LM diagnosis and supportive palliative care was only decided on for two patients, due to rapid disease progression or poor performance. Treatment for LM included systemic therapy in 27 patients (93%) and combined RT in 9 patients (31%).

Combined RT was SRS in seven patients (78%) and WBRT in two patients (22%). No additional specific toxicity related to RT was noted, including when administered concurrently with systemic therapy.

Among the 27 systemically treated patients after LM diagnosis, 17 patients (59%) were treated with immunotherapy (5 with ipilimumab, 7 with anti-PD1 and 5 with nivolumab + ipilimumab), 5 patients (17%) received targeted therapy (BRAF inhibitor alone for 3 patients and BRAF + MEK inhibitors for 2 patients), 1 was treated with chemotherapy (temozolomide), and 4 patients (14%) were treated with anti-PD1 in combination with BRAF inhibitor.

### 3.4. Survival and Response Rate

**Figure 2:**
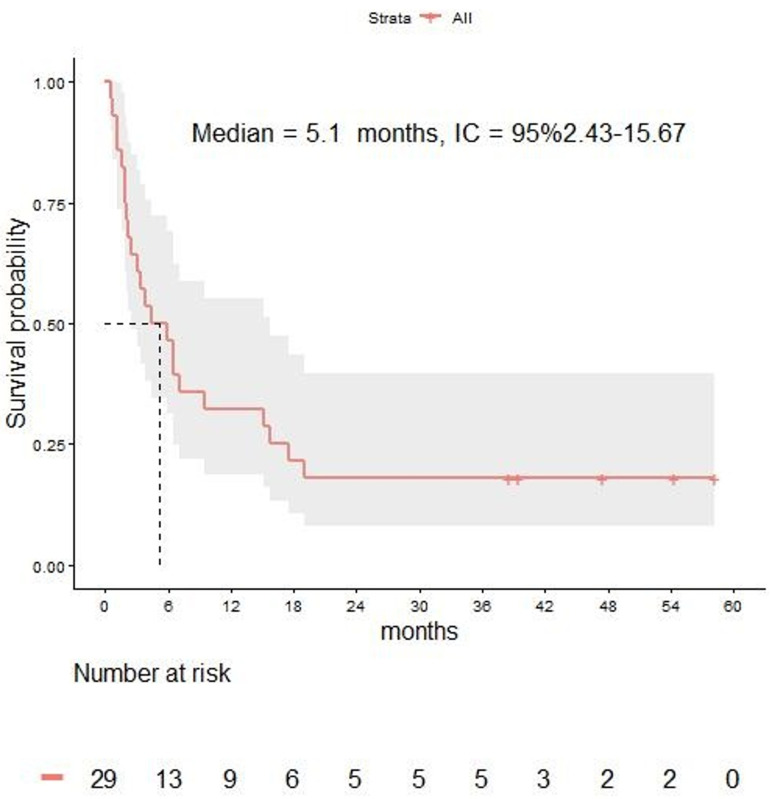
Overall survival in melanoma patients with leptomeningeal disease.

With a median follow-up of 4.4 months (range 1.9–15.7), the median OS from LM diagnosis was 5.1 months (95% CI 2.4–15.6) in the overall population (Figure 2). The 3-, 6-, 12-, and 24-month OS rates were 64%, 46%, 32%, and 18% respectively.

Median OS of the 10 patients in which treatment sequence after LM diagnosis included BRAF inhibitors was 6.4 months (3.3-not reached), while median OS was 5.1 months (2.4–18.9) for the 22 patients in which treatment sequence after LM diagnosis included immune checkpoint inhibitors.

Median OS was 7.1 months (range 3.8 to not reached) for the 9 patients receiving cRT and 3.2 months (2.1–15.1) for the 20 patients not receiving cRT.

**Figure 3:**
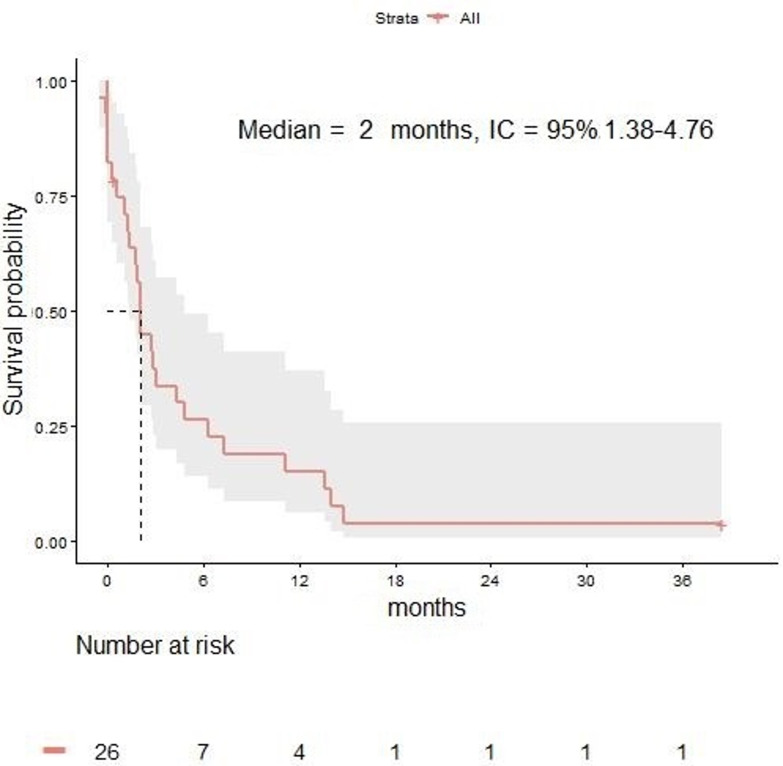
Progression-free survival in melanoma patients with leptomeningeal disease.

The median PFS from LM diagnosis was 2 months (95% CI 1.3–4.7) in the overall population (Figure 3). The 3-, 6-, 12-, and 24-month OS rates were 34%, 26%, 15%, and 4% respectively.

In the total population, the ORR was 17% (IC ORR of 17% and EC ORR of 34%). The IC ORR and EC ORR were 22% and 44% in patients receiving RT versus 5% and 30% in patients not receiving RT, respectively.

### 3.5. Analysis of Factors Affecting Survival

Results of univariate and multivariate analysis of potential prognostic factors are listed in Table 2. Only factors for which adequate numbers of data points were available are included in the analysis. The number of data points was not sufficient to assess the impact of cRT on OS. Age (HR 0.99, 95% CI 0.96–1.02, *p* = 0.54), sex (HR 0.86, 95% CI 0.37–1.92, *p* = 0.72), BRAF mutation status (HR 0.53, 95% CI 0.23–1.21, *p* = 0.13), and ECOG (HR 1.46, 95% CI 0.64–3.35, *p* = 0.37) did not have a significant impact on OS. There were no differences in OS in patients who had MBM at LM diagnosis versus those without (HR 1.52, 95% CI 1.14–1.95, *p* = 0.3). Immunotherapy was not associated with significantly improved OS (HR 1.45, 95% CI 0.64–3.29, *p* = 0.37).

Clinical symptoms at LM diagnosis was associated with significantly shorter overall survival in univariate analysis (HR 2.96, 95% CI 1.25–6.99, *p* = 0.01) but not in multivariate analysis (HR 2.35, 95% CI 0.9–6.1, *p* = 0.08). Median OS was 3.12 months (2.07–7.13) in patients with neurological symptoms at LM diagnosis compared to 12.3 months in patients without neurological symptoms (*N* = 5.91-NA).

An elevated serum LDH level was associated with significantly shorter overall survival in univariate (HR 1.44, 95% CI 1.09–1.90, *p* < 0.01) and multivariate analysis (HR 1.52, 95% CI 1.14–1.95, *p* < 0.01).

Median OS was 3.0 months (1.2-NR) in patients with neurological symptoms and elevated serum LDH at LM diagnosis (*N* = 7), compared to 16.33 months in patients without neurological symptoms and normal serum LDH at LM diagnosis (*N* = 12).

### 3.6. Characteristics of Alive Patients at Time of Data Analysis

At time of data analysis (May 2020), five patients are still alive and had complete response at the last imaging evaluation dating less than 3 months (Table 3).

The median follow-up of alive patients at time of analysis is 47.4 months (range 39.4–54.3). Among five patients alive at time of analysis, only one was treated with BRAF and MEK inhibitors and four have no treatment.

All of them had good prognostic factors at LM diagnosis, namely ECOG 0 or 1, normal LDH level and absence of neurological symptoms. Interestingly, each of them had received combined radiotherapy with anti-PD1 +/− anti-CTLA4 at some point in their disease course. Treatment discontinuation for immune-related grade 3 or 4 adverse events was observed in patients 2 and 3.

Patient one presented initially a solitary MBM, treated with surgery and adjuvant radiotherapy of the post-operative cavity. Two months later, he developed asymptomatic LM and lymph nodes, pulmonary, and liver metastases. Nivolumab and ipilimumab therapy was started in combination with WBRT. Grade 3 hepatitis required oral corticosteroids and temporary immunotherapy discontinuation. WBRT was complicated by radionecrosis two years later. Immunotherapy was discontinued since June 2019 and patient is still in complete response at the last follow-up.

Patient two was diagnosed with stage IV melanoma with concomitant LM and MBM and lymph nodes, cutaneous, and pulmonary involvement 9 months after the diagnosis of primary melanoma. She was treated with nivolumab and ipilimumab combined with SRS. Five months after the initiation of systemic treatment, MRI revealed new nodular leptomeningeal enhancement which were treated with SRS. Anti-PD1 antibody was continued for three years with persistent intra-cranial and extra-cranial complete response until she presented immune-related grade 4 eosinophilic fasciitis-like toxicity requiring high dose corticosteroids and immunotherapy cessation. Fourteen months after immunotherapy cessation, the patient is still in complete response.

Patient five was treated with dabrafenib alone for advanced melanoma, without CNS involvement for 10 months, when asymptomatic LM and MBM were diagnosed on systematic MRI, with concomitant extra-cranial complete response. Treatment with anti-PD1 and SRS was performed but grade 3 pulmonary adverse event three months later required immunotherapy cessation. Dabrafenib was reintroduced in association with trametinib. Four months after the initiation of targeted therapy, the patient was in complete intra- and extra-cranial response which was maintained for 43 months at the last follow-up.

## 4. Discussion

This study confirmed the poor prognosis of melanoma patients with LM but brought some hope for the future in the light of survival benefit observed in the era of immunotherapy and targeted therapy. The median OS of 5.1 months described in this study compares favorably to reported outcomes before the arrival of targeted therapy and checkpoint inhibitors, when chemotherapy was the only available systemic option [2,3,4] (Table 4). In 2008, Harstad et al. described a large cohort of 110 melanoma patients with LM treated between 1944 and 2002 and reported a median OS of only 10 weeks [3]. To date, few retrospective studies have evaluated the outcomes of melanoma patients with LM treated with new targeted and immune therapies [11,12,13]. Notably no or few patients included in these studies were treated with anti-PD1 [11,12,13]. First, Geukes Foppen at al. reported the outcomes of 39 melanoma patients with LM treated between 2010 and 2015 [12]. The median OS of the 24 patients treated with ipilimumab or BRAF inhibitors was 21.7 weeks, while the median OS of untreated patients was 2.9 weeks [12]. In a case series of 14 melanoma patients with LM reported by Arasaratnam et al., the median OS of patients receiving ipilimumab, anti-PD1 and BRAF inhibitors were 3 months, 7.1 months, and 7.2 months respectively [11]. In 2019, Ferguson et al. reported the largest retrospective cohort of melanoma patients with LM (*N* = 178) with a median OS of 2.9 and 8.2 months in patients treated with immunotherapy (*N* = 12) and targeted therapy (*N* = 60), respectively [13]. Notably, all patients included in this study were diagnosed with LM between 1999 and 2015 and, therefore, before the approval of anti-PD1 monoclonal antibodies, and the association of nivolumab and ipilimumab. Therefore, our study represented the largest cohort of melanoma patients with LM treated with immunotherapy reported to date.

Interestingly, the median OS of patients receiving cRT was numerically higher than those in patients not receiving cRT (7.13 months and 3.22 months respectively) but statistical analyses were not allowed as the number of data points was not sufficient to assess the impact of cRT on OS. However, this observation should be interpreted with caution due to some confounding bias: the small number of patients in each group, the retrospective analysis of data, and the selection by clinicians of patients with better performance status, which were more likely to receive a combined approach. To the best of our knowledge, no trial has investigated the impact of RT timing with new systemic treatment in melanoma patients with LM. Some evidence exists regarding the interest of SRS to treat focal LM and to control neurological symptoms [5,14]. RT combined with systemic treatment has recently demonstrated a survival benefit in 262 patients with MBM with a significant decrease of 40% in the risk of death [15]. Notably, 10% of patients had both LM and MBM. Given the presence of confounding bias in our study, we cannot judge the effectiveness of combined radiotherapy and new systemic treatment. However, this observation provides data to support the rationale for clinical studies assessing a combined approach included SRS and modern systemic therapy for melanoma patients with LM.

A very encouraging finding of this study is the identification of long-term survivor patients. Among 29 patients analyzed, five are still alive at time of analysis, with a median follow-up of 47.4 months, and had long-term complete response. Despite the overall poor prognosis, a subset of melanoma patients with LM could achieve longer survival [12,13]. Some factors commonly associated with improved survival are actually known [3,12,13]. Ferguson et al. have demonstrated several factors significantly associated with improved survival on multivariate analysis included good ECOG score (ECOG = 0), absence of neurological symptoms, lack of concurrent systemic disease, no systemic therapy prior to LM diagnosis, treatment with targeted therapy, and intra-thecal therapy after LM diagnosis [13]. Previously, Harstad et al. revealed that a history of a primary melanoma lesion originating on the trunk predicted shorter survival after LM diagnosis, and intra-thecal chemotherapy predicted longer survival [3]. In this study, an elevated serum LDH level and the presence of neurological symptoms at LM diagnosis was associated with significantly shorter overall survival, which is consistent with previous reports [3,12,13]. However, none of the other analyzed factors (age, sex, BRAF mutation status, ECOG, presence of MBM, type of treatment) were found to be a significant predictor of survival, probably given to the small number of patients.

One of the strengths of this study is the central neuro-oncologists’ reviewing of MRI findings according to the EANO-ESMO guidelines. Although CSF analysis is the gold-standard, MRI is now the initial, and often sole, tool to diagnose LM [17,18]. This non-invasive imaging method could be routinely performed in melanoma patients to early diagnose a leptomeningeal involvement, and has a sensitivity and predictive positive value equal to CSF analysis [17,18].

We are aware that our study has some limitations. The small number of patients and the retrospective analysis of data will generate hypotheses to be tested in larger prospective studies.

This study provides evidence of the urgent need for more effective therapies for patients with LM from melanoma. Multiple clinical trials have shown encouraging results in MBM patients treated with immunotherapy and targeted therapy [6,7,8,9,10]. However, all these trials excluded patients with LM due to the overall poor prognosis. Survival data described in this study in the era of modern therapies and the identification of long-term survivors, support the rationale for therapeutic trials in this under-studied population. A phase I trial investigating concurrent intravenous and intrathecal nivolumab in melanoma patients with LM is currently ongoing (NCT03025256, https://clinicaltrials.gov/ct2/show/NCT03025256), with encouraging preliminary safety data.

## 5. Conclusions

Despite many advances in advanced melanoma treatment, patients with LM from melanoma still have an extremely poor prognosis. Fortunately, new treatment modalities, such as targeted therapy or immune checkpoint inhibitors and combined radiotherapy, appear to be promising new treatment options for melanoma LM, and need to be tested in larger prospective studies. A subset of patients are long-term survivors, and factors associated with improved survival such as low serum LDH level may be identified.

## Figures and Tables

**Table 1:** Characteristics of melanoma patients at leptomeningeal disease diagnosis.

Characteristics		Number of Patients (%)
Median Age: 55 Years (Range: 50–67)		
Sex	Female	11 (38%)
	Male	18 (62%)
ECOG	0	12 (42%)
	1	10 (34%)
	2	4 (14%)
	3	2 (7%)
	4	1 (3%)
Serum LDH	Normal	20 (69%)
	Elevated	9 (31%)
BRAF	Wild type	15 (52%)
	Mutated	13 (45%)
	NA	1 (3%)
NRAS	Wildtype	15 (52%)
	Mutated	11 (38%)
	NA	3 (10%)
Primary melanoma site	Trunk	6 (22%)
	Arms	7 (24%)
	Leg	3 (10%)
	Acral lentiginous	5 (17%)
	Head	1 (3%)
	NA	7 (24%)
MBM at LM diagnosis	Yes	19 (66%)
	No	10 (34%)
Number of MBM at LM diagnosis	1	5 (18%)
	2–4	10 (35%)
	5–9	3 (10%)
	>10	1 (3%)
Size of MBM	<2 cm	13 (45%)
	>2 cm	6 (21%)
Corticosteroids	Yes	7 (24%)
	<0.5 mg/kg/day	4 (14%)
	>0.5 mg/kg/day	3 (10%)
Previous radiotherapy for MBM	No	12 (42%)
	SRS	10 (34%)
	WBRT	4 (14%)
	SRS and WBRT	3 (10%)
Previous surgical resection of brain metastases	Yes	5(17%)
	No	24 (83%)
Number of previous systemic line	0	14 (48%)
	1	6 (21%)
	2	5 (17%)
	3	2 (7%)
	4	2 (7%)
Neurological symptoms	Yes	15 (52%)
	No	14 (48%)
CSF cytology	Negative	1 (3%)
	Positive	1 (3%)
	NA	27 (94%)
Radiographic findings of LM	Yes	29 (100%)
	No	0 (0%)

CSF: cerebrospinal fluid; ECOG: Eastern Cooperative Oncology Group; NA: data unavailable; LM: leptomeningeal metastases; MBM: melanoma brain metastases; SRS: stereotactic radiosurgery; WBRT: whole brain radiation therapy.

**Table 2:** Univariate and multivariate analysis: factors associated with overall survival after diagnosis of leptomeningeal disease.

Analyzed Factors	Univariate Analysis		Multivariate Analysis ***	
	HR (95% CI)	*p* Value	HR (95% CI)	*p* Value
Treatment **	1.45 (0.64–3.29)	0.37	NR	NR
Sex *	0.86 (0.37–1.97)	0.72	NR	NR
Age *	0.99 (0.96–1.02)	0.54	NR	NR
LDH *	1.44 (1.09–1.90)	<0.01	1.52 (1.14–1.95)	<0.01
ECOG *	1.46 (0.64–3.35)	0.37	NR	NR
BRAF *	0.53 (0.23–1.21)	0.13	0.45 (0.17–1.19)	0.23
MBM *	0.63 (0.27–1.51)	0.30	NR	NR
Neurological symptoms *	2.96 (1.25–6.99)	0.01	2.35 (0.9–6.1)	0.08

HR: hazard ratio; LDH: lactate dehydrogenase level; ECOG: Eastern Cooperative Oncology Group; MBM: melanoma brain metastases. * at LM diagnosis. ** systemic treatment (immunotherapy, targeted therapy, chemotherapy) received after LM diagnosis. *** Only variables with *p* value inferior or equal to 0.20 in univariate analysis were included in multivariate analysis.

**Table 3:** Characteristics of alive patients at the time of data analysis.

Patient	Age/Sex *	ECOG *	BRAF Status	LDH *	MBM	LM Symptoms	Method of Diagnosis	Radiology	Treatment Prior to LM Diagnosis	Time from Primary Melanoma to LM Diagnosis	Time from Metastatic Melanoma to LM Diagnosis	Systemic Treatment Sequence Post LM Diagnosis	Radiotherapy	Time from LM Diagnosis to Last FU	Severe AEs	Status
1	62 M	0	WT	N	Yes	No	MRI	Pachy/leptomeningeal enhancement	None	16 years	2 months	NIVO+IPI	SRS and WBRT	48 months	Radio necrosis	CR
2	52 F	0	WT	N	Yes	No	MRI	Nodular leptomeningeal enhancement	None	9 months	None	NIVO+IPI	SRS	45 months	Immune-related grade 4 eosinophilic fasciitis like toxicity	CR
3	69 M	0	WT	N	Yes	No	MRI	Nodular leptomeningeal enhancement	DCZ	None	3 months	1st: IPI 2nd: NIVO 3rd: NIVO + MEK inh	SRS	64 months	Immune-related grade 3 pulmonary toxicity	CR No treatment since 26 months
4	58 M	0	WT	N	Yes	No	MRI	Nodular leptomeningeal enhancement	IPI	14 months	6 months	NIVO	SRS	67 months	None	CR No treatment since 22 months
5	57 F	1	MT	N	Yes	No	MRI	Nodular leptomeningeal enhancement	BRAF inh	46 months	10 months	1st: PEMBRO 2nd: BRAF inh + MEK inh	SRS	64 months	None	CR Already treated with BRAF and MEK inh

* At LM diagnosis; M: male; F: female; N: normal; ECOG: Eastern Cooperative Oncology Group; WT: wild-type; MT: mutant; MBM: melanoma brain metastases; LM leptomeningeal disease; MRI: magnetic resonance imaging; DCZ: dacarbazine; NIVO: nivolumab; IPI: ipilimumab; PEMBRO: pembrolizumab; inh: inhibitors; SRS: stereotaxic radiosurgery; WBRT: whole brain radiotherapy; AEs: adverse events; FU: follow-up; CR: complete response.

**Table 4:** Previous studies evaluating melanoma patients with leptomeningeal disease.

Authors	Number of Patients, Time of Enrolment	Treatments	Median OS	Factors Associated with Survival in Multivariate Analysis
Ferguson et al.	*N* = 178 Between 1999 and 2015	> RT: *N* = 98	All patients: 3.5 months Untreated patients: 0.7 months Any treatment: 4.4 months	
		> Systemic therapy: *N* = 11 - Targeted therapy: *N* = 60 - Immunotherapy: *N* = 12 - Chemotherapy: *N* = 89 - Intra-thecal therapy: *N* = 64	Patients treated with: - RT: 4.6 months - Intrathecal therapy: 7.8 months - Immunotherapy: 2.9 months - Targeted therapy: 8.2 months - Chemotherapy: 4.7 months	Improved OS was associated with: - Good PS (ECOG 0) (HR 2.1, 95% CI 1.3–3.1, *p* = 0.001) - Lack of concurrent systemic disease (HR 0.4, 95% CI 0.3–0.8, *p* = 0.025) - Treatment with targeted therapy (HR 0.6, 95% CI 0.4–0.9, *p* = 0.006) or intra thecal therapy (HR 0.5, 95% CI 0.3–0.8, *p* = 0.002) after LM diagnosis Shorter OS was associated with: - Presence of neurological symptoms (HR 1.6, 95% CI 1.1–2.4, *p* = 0.001) - Any systemic therapy prior to LM diagnosis (HR 1.6, 95% CI 1.0–2.5, *p* = 0.05)
Arasaratnam et al.	*N* = 14 Between 2012 and 2015	> RT: *N* = 11	All patients: 5.2 months	NA
		> Systemic therapy: *N* = 11 - Targeted therapy: *N* = 4 - IPI: *N* = 2 - anti-PD1: *N* = 5 -IPI + NIVO: *N* = 1	Patients treated with: - IPI: 3 months - anti-PD1: 7.1 months - BRAF inh: 7.2 months	
Geukes Foppen et al.	*N* = 39 Between 2010 and 2015	> RT: *N* = 15	Untreated patients (*N* = 14): 2.9 weeks	Shorter OS was associated with elevated serum LDH (*p* < 0.001) and S100B (*p* = 0.04) at LM diagnosis
		> Systemic therapy: *N* = 21 - included BRAF inh: *N* = 14 - included IPI: *N* = 10 - included anti-PD1: *N* = 0	Treated with IPI or BRAF inh (*N* = 24): 21.7 weeks	
Hastad et al.	*N* = 110 Between 1994 and 2002	> RT: *N* = 48 > Chemotherapy: *N* = 42 > Intrathecal therapy: *N* = 53	10 weeks	Improved OS was associated with intra-thecal chemotherapy (HR = 0.5, 95% CI 0.4–0.8, *p* = 0.0036)
Pape et al.	*N* = 9 Between 2007 and 2011	> Combination of intra-thecal chemotherapy and systemic chemotherapy: *N* = 9	8 weeks	NA
Raizer et al.	*N* = 40 Between 1991 and 2001	> Systemic chemotherapy +/− RT: NA	4 months	NA

RT: radiotherapy; OS: overall survival; PS: performance status; ECOG: Eastern Cooperative Oncology Group; NA: data unavailable; IPI: ipilimumab; inh: inhibitors; NIVO: nivolumab; N: number of patients; HR: hazard ratio.
